# Novel role of the SIRT4-OPA1 axis in mitochondrial quality control

**DOI:** 10.15698/cst2018.01.118

**Published:** 2017-12-23

**Authors:** Alexander Lang, Roland P. Piekorz

**Affiliations:** 1 Institut für Biochemie und Molekularbiologie II, Medizinische Fakultät der Heinrich-Heine-Universität, Düsseldorf, Germany.

**Keywords:** mitochondrial quality control, mitophagy, senescence, aging, SIRT4, OPA1

## Abstract

Mammalian sirtuins are fundamental regulators of a plethora of cellular functions, including gene expression, proliferation, metabolism, and ultimatively cellular aging and organismal life-span. The mitochondrial sirtuin SIRT4 acts as metabolic tumor suppressor and is down-regulated in many cancer types. We showed that SIRT4 expression was up-regulated during replicative senescence and by different anti-proliferative and senescence inducing stressors, including UVB and ionizing radiation, due to inhibition of its negative regulator, microRNA miR-15b. In our recent studies we addressed the molecular consequences of increased SIRT4 expression for mitochondrial function and quality control. We demonstrated that SIRT4 reduces O_2_ consumption and decreases mitochondrial membrane potential in line with an increased generation of mitochondrial reactive oxygen species (mtROS). This led to the accumulation of dysfunctional mitochondria and a more fused mitochondrial network associated with a decreased mitophagic clearance. Mechanistically, our data indicate that SIRT4 promotes mitochondrial fusion in an enzymatically dependent manner through interaction with and stabilization of the dynamin-related GTPase L-OPA1, thereby opposing fission and mitophagy. Our findings provide novel insight in the role of SIRT4 as stress triggered factor that causes mitochondrial dysfunction and impaired mitochondrial quality control through decreased mitophagy, a major hallmark of aging.

Aging is defined by various molecular and cellular hallmarks, among them stem cell exhaustion, genomic instability, altered proteostasis, cellular senescence, and mitochondrial dysfunction 1. Mitochondrial defects are considered major drivers of cellular aging and are characterized by mtDNA mutations, respiratory chain dysfunction and increased mtROS generation, or altered mitochondrial quality control through impaired clearance of dysfunctional mitochondria through selective autophagy (mitophagy) 2,3,4. A major group of proteins implicated in the regulation of aging processes are sirtuins which represent NAD^+^-dependent enzymes with a variety of enzymatic activities, substrates, and cellular functions 5. Among the sirtuins, SIRT3, SIRT4, and SIRT5 define a subgroup based on their localization in mitochondria where they control various metabolic pathways, including the respiratory chain 6, β-oxidation of fatty acids 7, and the urea cycle 8.

SIRT4, that besides other targets, inhibits PDH (pyruvate dehygrogenase) 9 and GDH (glutamate dehydrogenase) 10 as key anaplerotic enzymes feeding into the citric acid cycle, represents a metabolic tumor suppressor that is down-regulated in many cancer types. We have shown that various senescence inducing stress stimuli, including UVB and ionizing γ-radiation, as well as replicative senescence, up-regulate SIRT4 expression due to inhibition of its negative regulator microRNA miR-15b that is downregulated in various senescence models 11. *In vivo*, we demonstrated an inverse correlation between miR15b and SIRT4 expression in photoaged vs. non-photoaged human skin. The senescence associated role of the miR-15b - SIRT4 axis was further strengthened by the observation that oligonucleotide mediated inhibition of miR‐15b altered, in a SIRT4-dependent manner, the expression of components of the senescence associated secretory phenotype (SASP; 12) and nuclear encoded mitochondrial genes, including TFAM (mitochondrial biogenesis; 13) and NRF1 (antioxidant response; 14) 11.

Based on further work we suggest that the SIRT4-OPA1 axis represents a major effector mechanism responsible for the aging associated decrease in mitophagy. Besides impacting mitochondrial function SIRT4 also alters mitochondrial quality control. Our findings indicate that elevated SIRT4 levels impact on the respiratory chain (presumably via interaction with components of complexes I and/or V) associated with reduced O_2_ consumption under basal and mitochondrial stress conditions, drop in mitochondrial membrane potential (Δ(m), and increased mitochondrial ROS (mtROS) levels 15. As a consequence, we detected an increase in mitochondrial mass that could be due to a compensatory increase in mitochondrial biogenesis and/or the accumulation of dysfunctional mitochondria due to impaired mitophagy. Contrary to the latter, autophagic flux, as determined by increased LC3B-II levels under mitochondrial stress conditions, was elevated in SIRT4 overexpressing cells, indicative of a higher autophagic rate to clear depolarized/dysfunctional mitochondria. However, further experiments revealed that the recruitment of the E3 ligase and mitophagy regulator Parkin to damaged mitochondria was decreased by approximately 40% when corrected for the almost 3-fold increase in mitochondrial mass 15. These findings are mechanistically supported by our observation that SIRT4 interacts with and stabilizes, in an enzymatically dependent manner, the dynamin related GTPase and major mitochondrial fusion promotor L-OPA1 (long form of OPA1), therefore negatively regulating mitophagy 15. In contrast, the proteolytically cleaved short form of OPA1, S-OPA1, promotes mitochondrial fission 16 and hence mitophagy. In line with our findings, SIRT4 was also shown to reduce ERK mediated phosphorylation of the pro-fission factor DRP1 thereby inhibiting mitochondrial fission 17 and presumably mitophagy. Taken together, increased SIRT4 expression seems not only to contribute to the pool of dysfunctional and more fused mitochondria, but also to inhibit their clearance by mitophagy.

In summary, our current model for the function of the SIRT4-OPA1 axis in mitochondrial fusion/fission regulation and mitochondrial quality control is summarized in **Figure 1**. The nature of the SIRT4-OPA1 interaction, the involved enzymatic activity of SIRT4 (ADP-ribosyltransferase 10, lipoamidase 9, or lysine deacylase 18) required for L-OPA1 stabilization or decreased proteolytic L-OPA1 processing (e.g. via the stress responsive mitochondrial protease OMA1), and the interaction of SIRT4 with mitophagy pathways (receptor mediated, Pink-Parkin driven) 19 are currently unclear. Moreover, SIRT4 may trigger the "retrograde" signalling response from mitochondria to the nucleus that is mediated by the AMPK/PGC1α pathway 20 and potentially contributes to the increased mitochondrial mass observed in SIRT4 overexpressing cells. Although our work was based on cell culture systems including primary human dermal fibroblasts, further studies will test the general significance and potential druggability of the miR-15b - SIRT4 - OPA1 axis *in vivo* to better understand and prevent stress-induced decrease in mitophagy as emerging effector of cellular aging.

**Figure 1:**
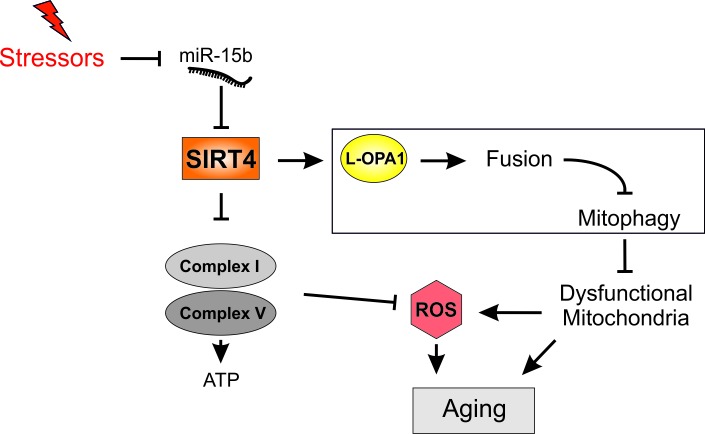
FIGURE 1: Illustration of the role of the SIRT4-OPA1 axis in mitochondrial quality control, mitophagy inhibition, and accumulation of functionally impaired mitochondria linked to cellular aging. The depicted interaction partners of SIRT4, including components of the complexes I and V of the respiratory chain, have been identified by mass spectrometric analyses (unpublished results). L-OPA1, long form of the dynamin-related GTPase and mitochondrial fusion regulator optic atrophy 1; ROS, reactive oxygen species.
